# ProdMX: Rapid query and analysis of protein functional domain based on compressed sparse matrices

**DOI:** 10.1016/j.csbj.2020.10.023

**Published:** 2020-11-24

**Authors:** Visanu Wanchai, Intawat Nookaew, David W. Ussery

**Affiliations:** Arkansas Center for Genomic Epidemiology & Medicine and The Department of Biomedical Informatics, University of Arkansas for Medical Sciences, Little Rock, AR 72205, USA

**Keywords:** Proteins, Protein functional domain, Domain architecture, Comparative genomics, Python, Compressed sparse matrix

## Abstract

Large-scale protein analysis has been used to characterize large numbers of proteins across numerous species. One of the applications is to use as a high-throughput screening method for pathogenicity of genomes. Unlike sequence homology methods, protein comparison at a functional level provides us with a unique opportunity to classify proteins, based on their functional structures without dealing with sequence complexity of distantly related species. Protein functions can be abstractly described by a set of protein functional domains, such as PfamA domains; a set of genomes can then be mapped to a matrix, with each row representing a genome, and the columns representing the presence or absence of a given functional domain. However, a powerful tool is needed to analyze the large sparse matrices generated by millions of genomes that will become available in the near future. The ProdMX is a tool with user-friendly utilities developed to facilitate high-throughput analysis of proteins with an ability to be included as an effective module in the high-throughput pipeline. The ProdMX employs a compressed sparse matrix algorithm to reduce computational resources and time used to perform the matrix manipulation during functional domain analysis. The ProdMX is a free and publicly available Python package which can be installed with popular package mangers such as PyPI and Conda, or with a standard installer from source code available on the ProdMX GitHub repository at https://github.com/visanuwan/prodmx.

## 1 Introduction

The comparison of protein functional domains is an important task in bioinformatics [1]. The protein functional domain concept allows researchers to capture common function of proteins from distantly related genomes, which is often seen as a major challenge in traditional sequence-homology based methods [2]. A protein functional domain represents a discrete structural unit that can convey a particular function. The different combinations of these functional units, known as domain architectures, which can be used as abstract models to simplify functional complexity in a protein [3]. The conservation of residues in each protein functional domain is determined by selective pressure. Base on the amino acid sequence variations of proteins that have common function enables the construction of profile hidden Markov models [4] for different functional domains. Pfam [5] is a popular database started more than two decades ago, that collects a broad set of protein functional domains using the HMMER tool [6]. This database also provides a web-based tool to search for both protein functional domains and domain architectures within a given sequence.

However, the analysis of protein functional domains and domain architectures in large-scale comparisons is a challenging task for web-based applications, especially in the analysis of functional conservation involving the complexity of resource and data management. Such tool requires a critical feature that allow users to quickly customize sets of interesting functions or organisms based on problem sets. Early tools for domain architecture comparison, such as CDART [7] are often implemented as a web-based application, and is limited by the number of inputs. That is, comparing millions of proteins will be difficult with these tools. Moreover, standalone tools, such as fungidomDB [8], often require time and effort from non-programmer biologists for installation of dependencies and usages.

Here, we present the ProdMX tool, a standalone Python tool that empowers researchers to explore functional domains and domain architectures of proteins across genomes of interest. With the state-of-the-art matrix compression algorithm, the ProdMX can be applied in the variety of applications including the high throughput screening for pathogenicity of genomes. The tool aims to reduce time and the computational resource used to calculate a large matrix of all-to-all comparison of functional domains or domain architectures. The ProdMX enables researchers familiar with the command prompt or the Python programming language to rapidly analyze specificity or conservation of functional domains or domain architectures. The ProdMX accelerates the protein functional research by offering an intuitive tool that can handle a massive amount of proteins when the computational resource is limited.

## 2 Implementation

### 2.1 The ProdMX tool design

**Fig. 1:**
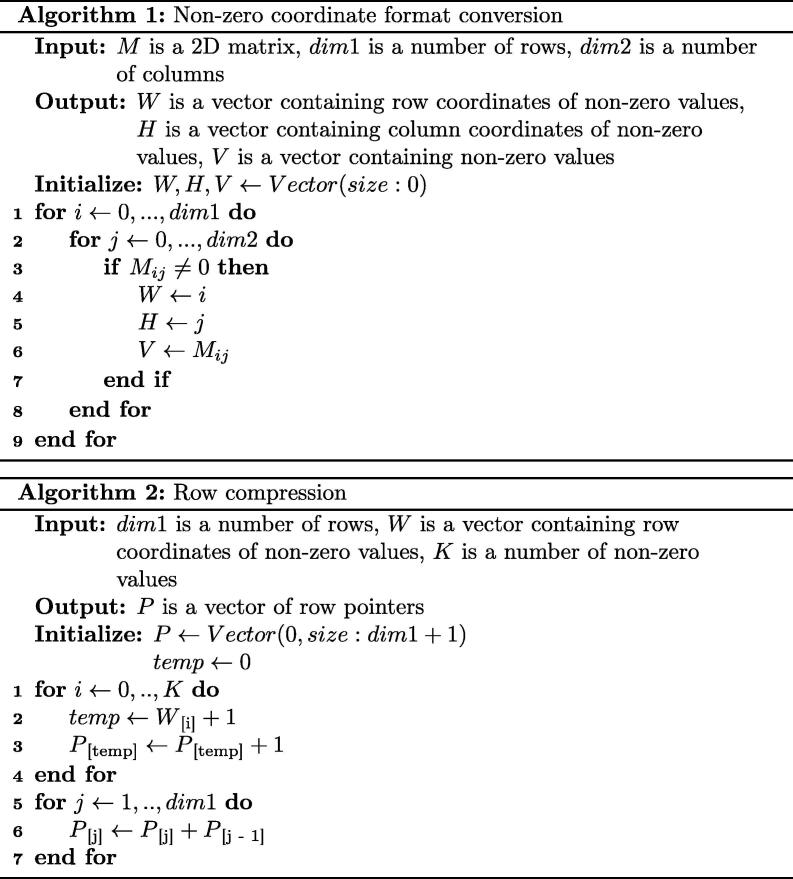
Algorithms for (1) conversion from a sparse matrix to coordinate form and (2) compression of row coordinates.

The ProdMX tool was designed to handle the large-scale analysis of both functional domains and domain architectures across million genomes. Due to the nature of the high complexity in the arrangement of protein functional domains in various genomes, the resulting matrix is both massive (challenging to compute) and sparse (that is, most cells are empty). The Compress Sparse Row (CSR) algorithm [9], [10] was introduced to reduce the sparse matrix size. This algorithm can handle both binary and non-binary matrix compression. The algorithm begins with a coordinate transformation of the sparse matrix to abolish zero values and store in row-column vectors and non-zero values. The row compression algorithm reduces the memory used for storing a vector of row by converting it into adjacent pairs of index pointers. In this way, the sparse matrix can be allocated in the computer’s local RAM, eradicating performance limitations due to input/output access boundaries of storage. The algorithms for sparse matrix conversion and row compression are shown in Fig. 1.

The data manipulation in the tool was handled with the Pandas package [11]. The database was implemented to store protein accessions associated with protein functional domains or domain architectures as an option for users with SQlite [12]. Generally, the use of the ProdMX tool starts with constructing the compressed sparse matrix in a command-line environment, using ProdMX as a Python package to load the matrix to the memory, and analyzing the number of conservations of either functional domains or domain architectures across sets of genomes, and generate the report of number or associated protein accessions. A complete description of all commands in the ProdMX is shown in Table 1.

**Table 1:** Utilities in the ProdMX tool.

Category/Utility	Description	Utility type	Input	Output
**Build matrix**				
prodmx-buildDomain	Build a folder containing a compressed sparse matrix of protein functional domains and index files	Command-line	[1]	[7]
prodmx-buildArchitecture	Build a folder containing a compressed sparse matrix of domain architectures and index files	Command-line	[1]	[7]

**Load matrix**				
loadMatrix	Load a compressed sparse matrix into an object variable	Package	[2]	[8]
loadBinMatrix	Load a binary compressed sparse matrix into an object variable	Package	[2]	[9]

**Analysis**				
getRow	Get a list of all row labels of the matrix	Package	–	[10]
getColumn	Get a list of all column labels of the matrix	Package	–	[11]
getProteinId	Get all protein id associated with given domain functional domains or domain architectures	Package	[3] (list_row), [4] (list_col), [5] (output)	[12]
sumRow	Summation of presence and absence values in row wise	Package	[3] (list_row), [4] (list_col)	[13]
sumColumn	Summation of presence and absence values in column wise	Package	[3] (list_row), [4] (list_col)	[14]
calCore	Calculate core protein functional domains or domain architectures	Package	[3] (list_row), [4] (list_col), [6] (counservation)	[15]

[1] a tab-delimited file of unique genome ids and hmmsearch result file paths, [2] a path to folder containing matrices and database from Prodmx's build matrix command,

[3] a list of genome ids, [4] a list of functional domains or domain architectures, [5] a result file path, [6] a conservation cut-off with a default at 95%,

[7] a folder containing compressed matrices and indexes of functional domains, [8] a ProdMX object for a count matrix, [9] a ProdMX object for a binary matrix,

[10] a list variable of all row labels (genome ids), [11] a list variable of all column labels (functional domain or domain architecture ids),

[12] a tab-delimited file of genome ids and protein ids,

[13] a Pandas dataframe of the count of domains or domain architectures for each genome,

[14] a Pandas dataframe of the count of genome for each domain or domain architecture,

[15] a pandas dataframe of the genome count for each core functinal domain or domain architecture.

**Fig. 2:**
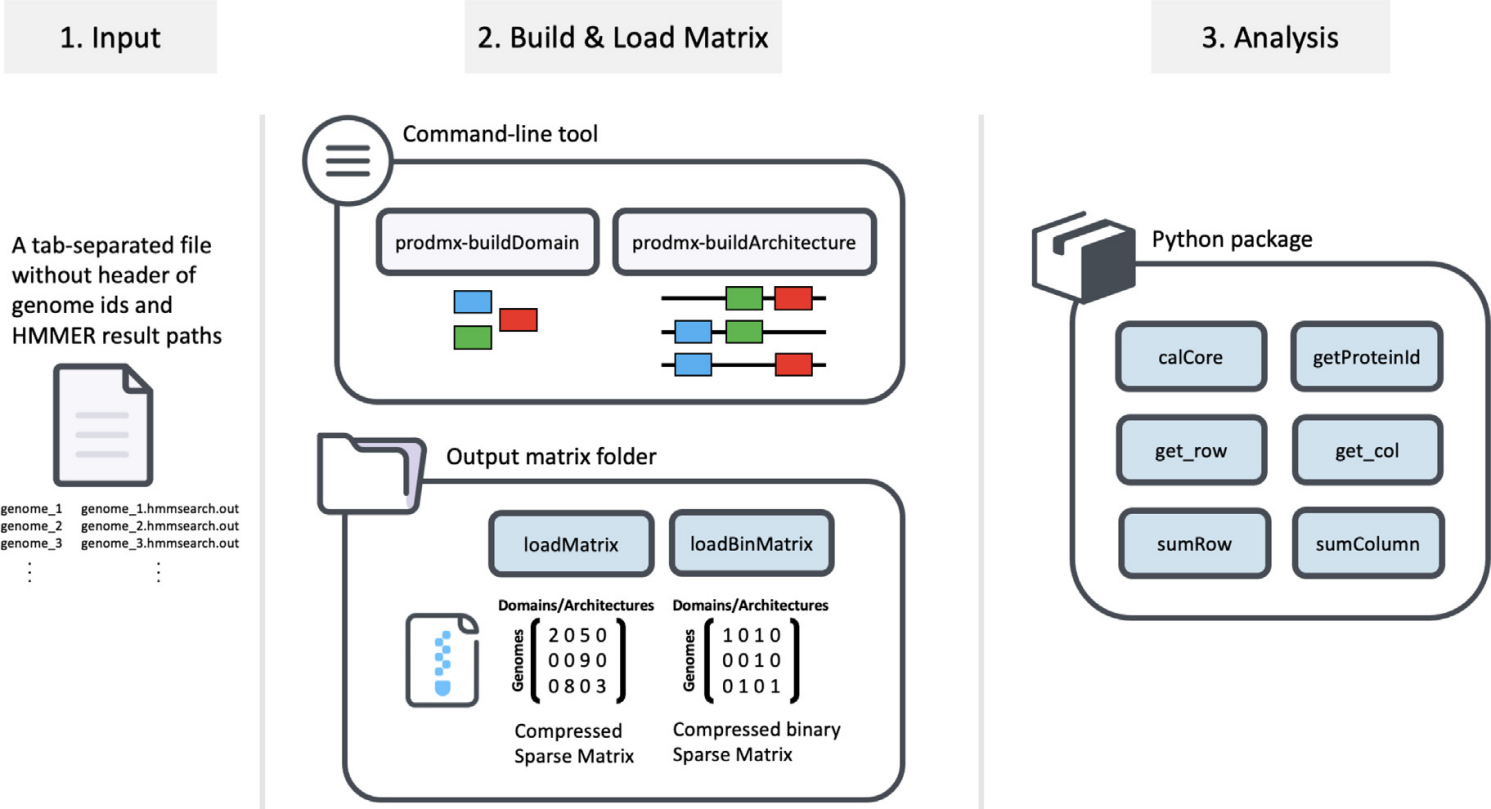
Structure and workflow of the ProdMX tool.

For the analysis and report generating parts, we designed the ProdMX tool to work as a Python package. This design allows users to flexibly use the ProdMX tool in the chain of commands with other tools in the user’s pipelines. In addition to this package design, users can quickly test the prototype of code or perform analyses on Jupyter Notebook, Python web-based interactive development environment [13]. The overview of the workflow is shown in Fig. 2.

### 2.2 Benchmarking

**Fig. 3:**
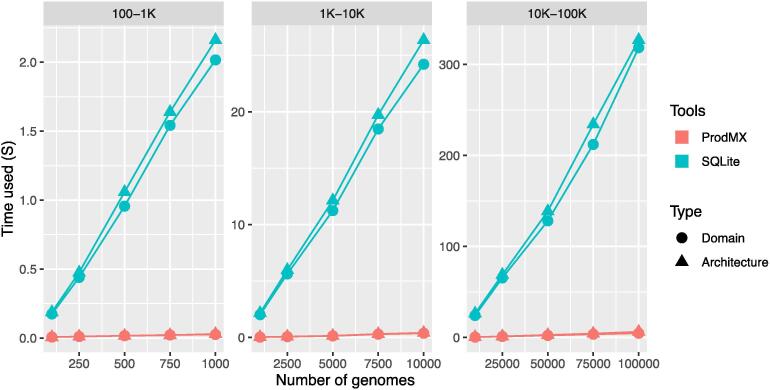
Performance benchmark of the average time of 100 replicates on analyses of core protein functional domains and core domain architectures using ProdMX and SQLite. Core functional domains and core domain architectures were retrieved using ProdMX and SQLite on different set of genomes ranging between 100–1000, 1000–10,000, and 10,000–100,000 genomes. The average query execution time of 100 replicates for each data set were collected.

**Fig. 4:**
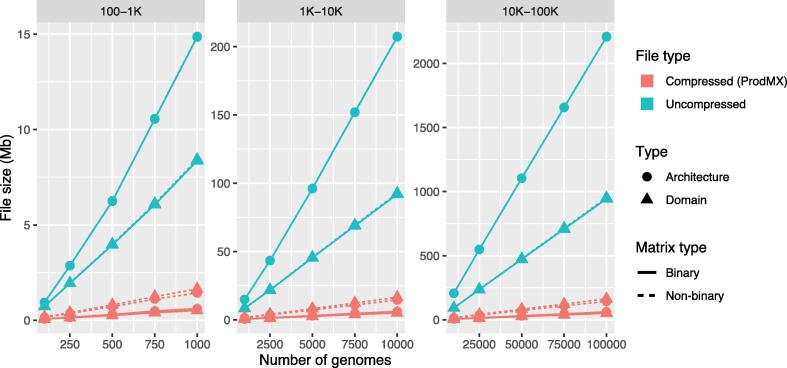
Sizes of storage occupied by protein functional domain and domain architecture matrices using ProdMX and plain text.

We performed benchmarking of the ProdMX tool using a set of 6881 high-quality *Escherichia coli* sequences, not derived from single cell nor metagenome project, having 0.8 or more for total and sequence quality scores in the dBBQs database [14], [15], on a single core of MacBook Pro 2.8 Ghz Intel i7, with 16 Gb of RAM. The genomes were run through Prodigal [16] for prediction of proteins; the proteins were then searched for functional domains using HMMER 3.1b2 with Pfam version 32 [17], resulting 4950 protein functional domains and 11,574 domain architectures. The HMMER results used in this benchmarking is available in the data availability statement section. To extend the number of genomes in the benchmarking sets, all sets of genomes were sampled from the same 6881 genomes. The compressed sparse matrices for functional domains and domain architectures were constructed for multiple sets of the good quality *E. coli* genomes (100–1000, 1000–10,000, and 10,000–100,000 genomes). These sets of genomes were also implemented in the database with a sparse structure in SQLite for retrieval speed comparison. We examined the average CPU runtime for calculating core functional domains and core domain architectures of 100 replicates for each set of genomes using a calCore utitity in ProdMX and SQLite queries (see supplementary file S2). Our benchmarking results show that the runtimes from ProdMX are approximately 63-fold faster than SQLite, and scale linearly with input size for functional domains and domain architectures (Fig. 3). For the compression ratio, we compared the size occupied by the compressed (ProdMX) and non-compressed with the same datasets used for the speed comparison. The results show that compression ratios of at least 8:1 for functional domain matrices and 17:1 for domain architecture matrices are achieved (Fig. 4). The database schemas representing protein functional domains and domain architectures, database queries, and codes for benchmarking can be found in the Supplementary Materials.

### 2.3 Installation and dependencies

The ProdMX requires an installation of Python 3.5 or newer, which is distributed through the Python Software Foundation [18]. Other dependencies can be detected and installed by either the Python Package Index (PyPI) or Conda [19]. The automated installation process from PyPI on different computer systems can be activated by executing: pip install prodmx. Also, the latest released version of ProdMX can be installed through conda:

conda install -c visanu prodmx.

Alternatively, the standard installation from ProdMX source code can be initiated via the pip installer:

python -m pip install /path/to/ProdMX_source_code.

We recommend users to take advantage of the automated installation methods from PyPI or Conda since they can precisely handle all different versions of dependencies on different system environments (Linux, Mac, and PC).

## 3 Usage and examples

### 3.1 Finding and analyzing of conservation of the region 2 domain of primary sigma factor (RpoD) across *Escherichia coli* genomes

**Fig. 5:**
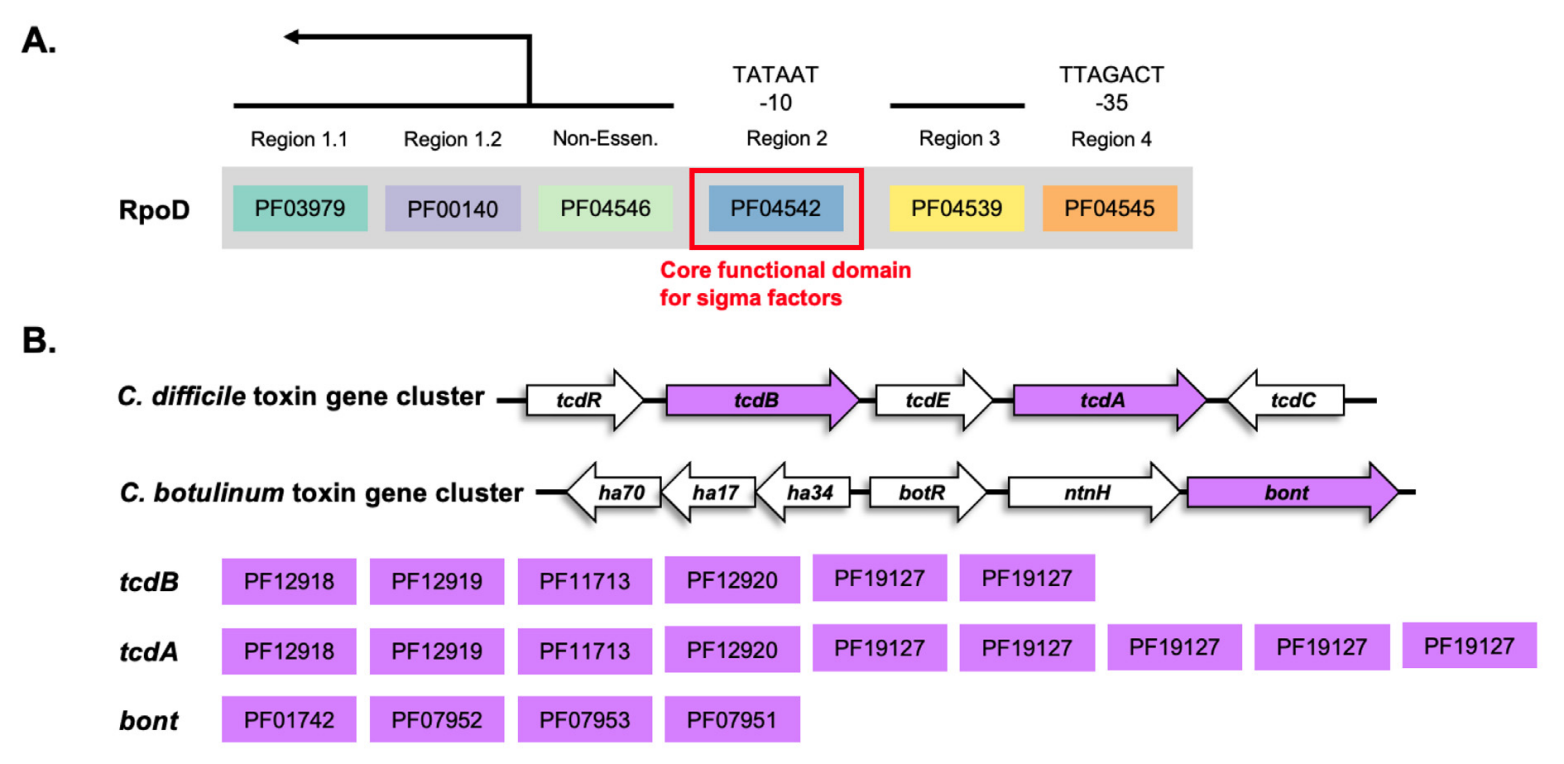
Genes and domain architectures for (A) primary sigma factor (RpoD) and (B) toxin genes in *C. difficile* and *C. botulinum*.

Sigma factors are proteins that regulate the transcription process by promoting binding of RNA polymerase to promoter sites of DNA sequence in prokaryotes [20]. Different groups of sigma factors are utilized to initiate different gene sets under different environmental conditions. Thus, analysis of diffrent groups of sigma factors allow us to identify types of regulon contributing to multiple functions of microbes, including virulence genes and virulence-associated genes. Here we show how to integrate the ProdMX to a pipeline for analyzing of the region 2 domain of primary σ^70^ protein (RpoD) [21] across high-quality *E. coli* genomes from the previous section (Fig. 5A). The following commands for the analysis pipeline of selected functional domain will be demonstrated in a Linux environment. The data used in the demonstration can be found in a test folder in the ProdMX repository.

First, we create a tab-delimited file as an input file for the tool. This input file includes two columns of genome labels and the path to their HMMER results. The output of the utility used in this step is the folder containing the compressed sparse matrix and its index file for the functional domain analysis. Also, a keep option (-k) allows us to store raw results of protein ids and their domains for further analysis. The example of command for building the sparse matrix folder in the command-line environment using the test data of high-quality *E. coli* genomes is shown below:

prodmx-buildDomain -i input_ecoli_id_hmm.tsv -o domain_matrix_fol -k

The ProdMX tool provides analysis utilities in the Python package for regular use and the interactive Python environment. In this demonstration, we choose to analyze our protein functional domain in Jupyter Notebook, the popular interactive Python environment. The binary matrix of presence and absence of each functional domain for each genome is then loaded to the memory by following commands:

import prodmx

import pandas as pd

binary_matrix = prodmx.loadBinMatrix(matrix_fol='domain_matrix_fol')

Further, to check if the region 2 domain of σ^70^ proteins (PF04542) is present in core functional domains (95% or more in conservation) across our set of *E. coli* genomes or not, we need to supply the calCore function with the information of list of genome labels and list of available protein functional domains as follows:

df_core_domain = binary_matrix.calCore(list_row=binary_matrix.getRow(), list_col=binary_matrix.getColumn())

Using a Pandas DataFrame command, we can select rows based on condition. The number of *E. coli* genome possessing the region 2 domain of σ^70^ (PF04542) can be retrieved by following command:

df_core_domain[df_core_domain['col_name'] == 'PF04542′]

Finally, the command below will be used to write all protein ids associated with the region 2 domain of σ^70^ for each genome to the output file in the tab-delimited format (GenomeId, Domain, ProteinId):

binary_matrix.getProteinId(list_row=binary_matrix.getRow(), list_col=['PF04542′], output='ecoli_domain_region2_RpoD_protein_id.txt')

### 3.2 Identifying and extracting of toxin genes from *C. difficile* and *C. botulinum* genomes

#### 3.2.1 Exotoxins from *C. difficile*

*Clostridioides difficile* or formerly known as *Clostridium difficile* is a group of bacteria that cause severe damage to the colon with diarrhea symptoms. This gram-positive species is one of the most common bacteria found in healthcare-associated infections (HAIs) in the United States [22]. The exotoxin gene cluster in *C. difficile* organize by two toxin genes *TcdA* and *TcdB* [23]. These two exotoxin genes are regulated by the alternative RNA polymerase sigma factor *TcdR* (Fig. 5A). To demonstrate the potential use case for screening, the ProdMX were employed to the identify the pathogenicity of unknown genome sequences from *Clostridiales* order.

Like the previous example, we need to go through the steps of creating a compress sparse matrix of the HMMER results for all genomes of interest. However, for the protein analysis, we need to construct the matrix of domain architectures since the order of functional domains within a protein can result in different gene function. The code to generate the compressed sparse matrix of domain architectures is as follows.

prodmx-buildArchitecture -i input_clostridiales_id_hmm.tsv -o architecture_matrix_fol -k

To count all virulence factors, the following codes were used to load the non-binary compressed sparse matrix of domain architectures to the python environment.

import prodmx

import pandas as pd

count_matrix = prodmx.loadMatrix(matrix_fol='architecture_matrix_fol')

To check for exotoxin genes, we retrieved the domain architectures from UniProt [24] for *TcdA* and *TcdB* protein*.* The dictionary between protein name and domain architectures were created as follows.

dict_tox = {'tcdB':'PF12918_PF12919_PF11713_PF12920_PF19127_PF19127′, 'tcdA':'PF12918_PF12919_PF11713_PF12920_PF19127_PF19127_PF19127_PF19127_PF19127′}

Using Pandas DataFame, we can create the data table for the in silico-screening of exotoxin in the unknown genomes by the code following:

list_result = []

for genome_id in count_matrix.getRow():

x = count_matrix.sumColumn(list_row=[genome_id], list_col=[dict_tox.get('tcdB'), dict_tox.get('tcdA')])['col_sum'].tolist()

list_result.append([genome_id]+x)

header=['genome_id', 'tcdB', 'tcdA']

pd.DataFrame(list_result, columns=header)

The table of genome and protein ids associating with exotoxins can be retrieved as follows:

count_matrix.getProteinId(list_row=count_matrix.getRow(), list_col=[ dict_tox.get('tcdB'), dict_tox.get('tcdA')], output='clostridiales_exotoxin_protein_id.txt')

#### 3.2.2 Neurotoxins from *C. botulinum*

The botulinum neurotoxins (BoNTs) produced by the strains of *Clostridium botulinum* can cause the disease botulism which is a potentially fatal disease in human [25]. This neurotoxin gene cluster in *C. botulinum* comprise of *ntnh* and *bont* genes with the alternative sigma factor *botR* to regulate the expression (Fig. 5B). Referring to the steps in the previous example of exotoxins, we can use the same domain architecture matrix to retrieve the potential genomes and protein ids that might associate with botulinum neurotoxins by following code:

dict_tox = {'bont': 'PF01742_PF07952_PF07953_PF07951′}

count_matrix.getProteinId(list_row=count_matrix.getRow(), list_col=[ dict_tox.get('bont'), output='clostridiales_neurotoxin_protein_id.txt')

The test data and extended versions for example 3.1 and 3.2 in Jupyter Notebook can be downloaded at the ProdMX GitHub repository (https://github.com/visanuwan/prodmx).

## 4 Conclusion

Here we introduce the ProdMX tool, which provides a native Python environment for analysis of the protein functional domain. While the functional domain analysis can be performed with the web-based applications, analyses of large queries are often limited by the bandwidth required to transfer data over the internet. Analyses with standalone tools are usually found to have insufficient memory issues due to the management of enormous sparse matrices and the complexity of dependencies required for tool installations. Overall results of benchmarking from ProdMX showed remarkably better performance over SQLite with sparse matrix schema implementation. With the provided use cases, ProdMX can be used as an effective tool for the high-throughput screening. We expect that the ProdMX tool will aid the scientific community in performing large queries and accelerate comparative genomics research that relies on accurate and clade-specific measures of protein functions.

## Data availability statement

Publicly available datasets were analyzed in this study. The data for benchmarking, list of genome assembly accessions, and HMMER results associated with the genomes from GenBank are available at https://app.box.com/s/n7chra1me0g5nv6yclzmshzns0drvxzo.

## Funding

This research was funded in part by the College of Medicine and the Department of Biomedical Informatics at UAMS, the Helen Adams & Arkansas Research Alliance Endowment. The National Science Foundation under Award No. OIA-1946391. National Institute of General Medical Sciences of the National Institutes of Health [P20GM125503] awarded to I.N.

## CRediT authorship contribution statement

**Visanu Wanchai:** Conceptualization, Software, Visualization, Writing - original draft, Writing - review & editing. **Intawat Nookaew:** Visualization, Validation, Writing - review & editing, Supervision. **David W. Ussery:** Supervision, Validation, Resources, Writing - review & editing.

## Conflict of Interest

The authors declare that the research was conducted in the absence of any commercial or financial relationships that could be construed as a potential conflict of interest.
