# Supplementary figures and images for: ProdMX: Rapid query and analysis of protein functional domain based on compressed sparse matrices
Source: Comput Struct Biotechnol J. 2020 Nov 24;18:3890–6. doi: 10.1016/j.csbj.2020.10.023 (PMC7719867; doi:10.1016/j.csbj.2020.10.023)

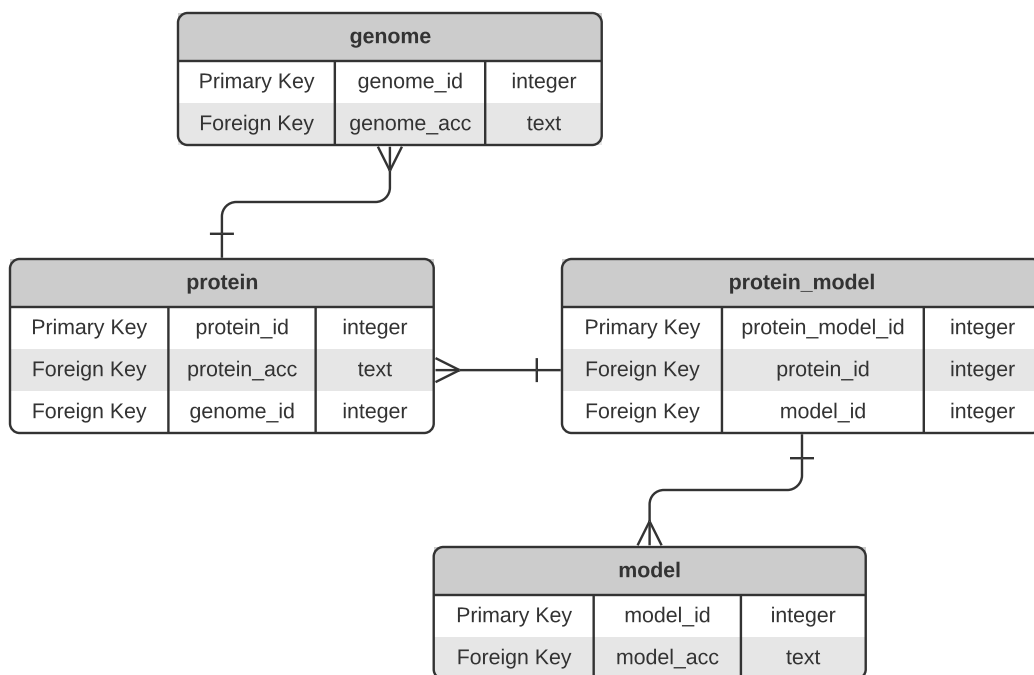

**Supplementary File S1 A database schema representing sparse matrix.**

Supplement: Supplementary Data 1 [file mmc1.pdf]
